# Role of N6-Methyladenosine (m^6^A) Methylation Regulators in Hepatocellular Carcinoma

**DOI:** 10.3389/fonc.2021.755206

**Published:** 2021-10-07

**Authors:** Nanfang Qu, Xiaotong Bo, Bin Li, Lei Ma, Feng Wang, Qinghua Zheng, Xuhua Xiao, Fengmei Huang, Yuanyuan Shi, Xuemei Zhang

**Affiliations:** ^1^Department of Gastroenterology, Affiliated Hospital of Guilin Medical University, Guilin, China; ^2^Department of Gastroenterology, The First Affiliated Hospital of Guangxi Medical University, Nanning, China; ^3^Department of Oncology, Affiliated Hospital of Guilin Medical University, Guilin, China; ^4^Department of Pathology, Affiliated Hospital of Guilin Medical University, Guilin, China

**Keywords:** hepatocellular carcinoma, N6-methyladenosine m^6^A, writers, readers, erasers

## Abstract

Liver cancer is the fifth most common malignant tumor in terms of incidence and the third leading cause of cancer-related mortality globally. Hepatocellular carcinoma (HCC) is the most common type of primary liver cancer. Although great progress has been made in surgical techniques, hepatic artery chemoembolization, molecular targeting and immunotherapy, the prognosis of liver cancer patients remains very poor. N^6^-methyladenosine (m^6^A) is the most abundant internal RNA modification in eukaryotic cells and regulates various stages of the RNA life cycle. Many studies have reported that the abnormal expression of m6A-related regulators in HCC represent diagnostic and prognostic markers and potential therapeutic targets. In this review, firstly, we introduce the latest research on m^6^A-related regulators in detail. Next, we summarize the mechanism of each regulator in the pathogenesis and progression of HCC. Finally, we summarize the potential diagnostic, prognostic and therapeutic value of the regulators currently reported in HCC.

## Introduction

Liver cancer is the fifth most common malignant tumor in terms of incidence and the third leading cause of cancer-related mortality globally (1). Among all primary liver cancers, hepatocellular carcinoma (HCC) is the most common, accounting for about 75–85% (1). Risk factors for liver cancer include viral infection (hepatitis B and C), fatty liver, heavy drinking, smoking, obesity, diabetes and aflatoxin contamination of food (2). Most patients are not candidates for curative treatments (such as surgical resection or liver transplantation) at diagnosis because of the extent or distribution of the tumor, underlying liver function, or medical comorbidities (3). The tyrosine multikinase inhibitors sorafenib and regorafenib were the first drugs approved as first- and second-line treatments, respectively, for advanced liver cancer. Although regorafenib can improve the survival rate (from 7.8 to 10.6 months) among patients whose tumors have progressed during sorafenib treatment, the side effects include impairment of liver function and performance status (PS) (4, 5). Worse still, the response rate among HCC patients to nivolumab, a programmed cell death protein-1 (PD-1) immune checkpoint inhibitor, is ≤20% (6). Thus, there is an urgent need for earlier diagnosis and new effective therapies.

Among the more than 170 types of RNA modification, m^6^A is the most abundant internal RNA modification in eukaryotic cells and it is related to almost every step of RNA metabolism (7). The modification is involved in all events in the entire life cycle of RNA molecules, including splicing, transportation, degradation, stability and translation (8). Consensus motif analyses revealed that m^6^A modification sites, known as DRACH motifs (R = G or A; H = A, C, or U; A is converted to m^6^A), in the transcriptome are not randomly distributed. Instead, they occur in coding sequences (CDS), 3’-untranslated regions (3’-UTRs) and the regions around stop codons (9, 10). RNA m^6^A modification is dynamically and reversibly regulated by methyltransferases (“writers”) and demethylases (“erasers”). These modifications are recognized by a group of binding proteins (“readers”) that recognize specific m^6^A-modified positions and subsequently regulate RNA functions (11).

The functions of m^6^A modification in mammals include the regulation of tissue development, circadian rhythm, DNA damage response, sex determination, T cell homeostasis and tumorigenesis (12, 13). There is a mounting number of studies showing that m^6^A dysregulation is critical in a variety of human cancers, including HCC (14–19). Furthermore, several m^6^A-related regulators have shown clinical value as biomarkers or therapeutic targets in HCC (20–24). Researches shown that m6A modification related to the etiology of HCC, including viral hepatitis and non- alcoholic fatty liver disease (NAFLD) (25).

Here, we summarized the physiological functions of m^6^A-related regulators and the potential role of m^6^A modification in HCC.

## Regulators of m^6^A Modification

**Figure 1:**
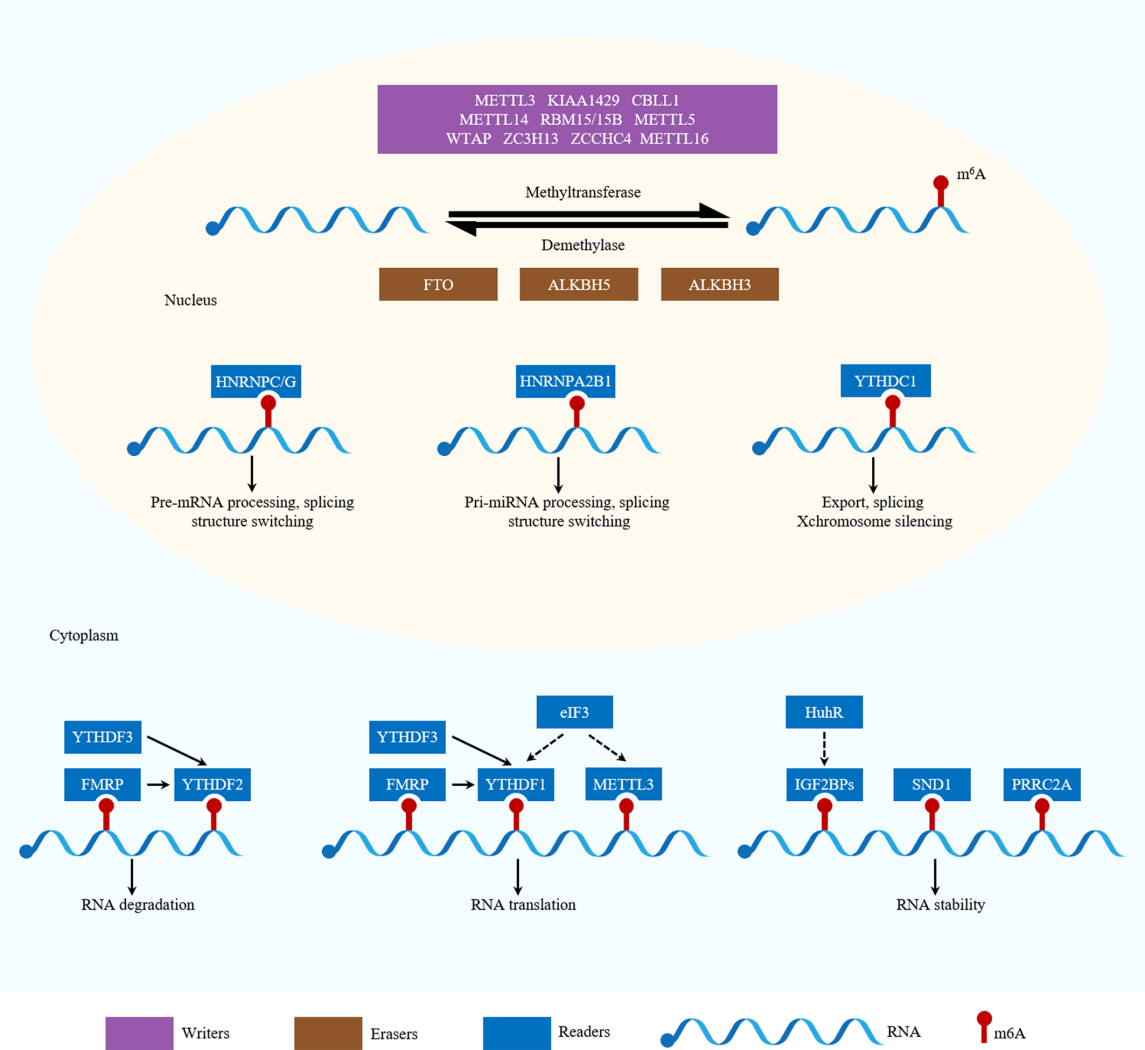
Functions of m6A-related regulators in RNA metabolism. m6A is catalyzed by writers and removed by erasers. METTL3, METTL14, WTAP, KIAA1429, RBM15/15B, ZC3H13, CBLL1, METTL5, ZCCHC4 and METTL16 are writers. ALKBH5, ALKBH3 and FTO are erasers. YTHDF1, YTHDF2, YTHDF3, YTHDC1, YTHDC2, hnRNPC/G, hnRNPA2B1, FMRP, eIF3, IGF2BPs, SND1 and PRRC2A are readers. Different readers binding to m6A sites can produce different biological effects.

The m^6^A-related regulators, comprising writers, erasers and readers, cooperatively maintain the dynamic and reversible balance of m^6^A methylation (12). Summaries of the functions of m^6^A-related regulators in RNA metabolism are shown in **Figure 1** and **Table 1**.

**Table 1:** Functions of m^6^A related regulators in RNA metabolism.

Categories	Regulator	Location	Function	Refs
**Writers**	METTL3	Nucleus/Cytoplasm	Catalyzes m^6^A modification and promotes translation by recruiting eIF3h to the translation initiation complex in cytoplasm	(26–28)
	METTL14	Nucleus	Stabilizes the METTL3-METTL14 complex	(22)
	WTAP	Nucleus	Recruits METTL3 and METTL14 to the nuclear speckle and regulates recruitment to mRNA targets	(29)
	KIAA1429	Nucleus	Mediates mRNA methylation and and associates with alternative polyadenylation	(30)
	RBM15/15B	Nucleus	Mediates m^6^A methylation of lncRNA XIST	(31)
	ZC3H13	Nucleus	Participates in controlling m^6^A modification	(32)
	CBLL1	Nucleus	Participates in controlling m^6^A modification	(32)
	METTL16	Nucleus	Functions as U6 snRNA methyltransferase and modification of mRNAs	(33, 34)
	METTL5	Nucleus	Modifications 18S rRNA	(35)
	ZCCHC4	Nucleus	Modifications 28S rRNA	(36)
	PCIF1	Nucleus	Catalyzes m^6^Am methylation	(37, 38)
	METTL4	Nucleus	Catalyzing m^6^Am methylation in U2 snRNA to regulate pre-mRNA splicing	(39)
**Erasers**	FTO	Nucleus	Removes the m^6^A modification	(40)
	ALKBH5	Nucleus	Removes the m^6^A modification	(41)
	ALKBH3	Nucleus	Removes the m^6^A modification of tRNA	(42, 43)
**Readers**	YTHDF1	Cytoplasm	Promotes translation by recruiting the eIF3 translation initiation complex	(44)
	YTHDF2	Cytoplasm/Nucleus	Promotes degradation in cytoplasm; binds m^6^A sites in 5’UTR and prevents demethylation by FTO in nucleus	(45, 46)
	YTHDF3	Cytoplasm	Promotes mRNA translation and degradation by interacting with YTHDF1 and YTHDF2 respectively	(47)
	YTHDC1	Nucleus	Regulates mRNA splicing, expediting mRNA export and is required for XIST-mediated transcriptional silencing	(31, 48, 49)
	YTHDC2	Cytoplasm/Nucleus	Regulates translation initiation and mRNA degradation	(50–52)
	HNRNPC	Nucleus	Regulates pre-mRNA processing, alternative splicing and acts as ‘‘m^6^A-switch’’	(53)
	HNRNPG	Nucleus	Modulates the alternative splicing and acts as ‘‘m^6^A-switch’’	(54)
	HNRNPA2B1	Nucleus	Stimulates microRNA processing, alternative splicing and acts as ‘‘m^6^A-switch’’	(55, 56)
	IGF2BP1/2/3	Cytoplasm	Enhances the stability and translation of their target mRNAs by recruiting RNA stabilizers, such as HuR	(57–63)
	FMRP	Cytoplasm	Represses translation and maintaining stability through interplaying with YTHDF1 and YTHDF2	(64, 65)
	LRPPRC	Cytoplasm	Maintains non-translated transcripts and regulates mitochondrial translation	(66, 67)
	eIF3	Cytoplasm	Enhances cap-dependent translation	(68)
	SND1	Cytoplasm	Stabilizes ORF50 RNA and essential for KSHV lytic replication	(69)
	PRRC2A	Cytoplasm	Stabilizes m^6^A-modified transcript Olig2	(70)
	Ribosome	Cytoplasm	Stagnates on mRNA containing m^6^A codon	(71)

### Writers

Writers, including methyltransferase-like 3 (METTL3), methyltransferase-like 14 (METTL14) and Wilm’s tumor 1-associated protein (WTAP), are the major components of the methyltransferase complex (MTC) in the nucleus, which is responsible for the m^6^A methylation process (11). METTL3 is the core catalytic enzyme for transferring methyl groups to N6 positions, while METTL14 is vital for structural stabilization of the METTL3–METTL14 complex and is involved in RNA substrate recognition (26). However, a few studies have shown that METTL3 can also act as an m^6^A cytoplasmic reader. METTL3 associates with ribosomes or directly and promotes the translation of certain mRNAs, including Epidermal growth factor receptor (EGFR) and the Hippo pathway effector Transcriptional co-activator with PDZ-binding motif (TAZ), independently of its methyltransferase activity or downstream m^6^A reader proteins (27). It does this by recruiting eukaryotic translation initiation factor 3 subunit H (eIF3h) to the translation initiation complex, thus promoting cancer cell growth, survival and invasion (28). As a regulatory subunit, WTAP ensures METTL3–METTL14 localization to the nuclear speckle and promotes catalytic activity. Additionally, WTAP may regulate MTC recruitment to mRNA targets (29).

Other identified writers include KIAA1429 (also known as Vir-like m^6^A methyltransferase-associated [VIRMA]), RNA-binding motif protein 15 and 15B (RBM15 and RMB15B), zinc finger CCCH domain-containing protein 13 (ZC3H13) and Cbl proto-oncogene-like 1 (CBLL1; also known as HAKAI). KIAA1429 preferentially mediates mRNA methylation in 3′-UTRs and near stop codons and is associated with alternative polyadenylation (30). RMB15 and RMB15B, two other WTAP interactors, can recruit the MTC to specific sites, thus mediating m^6^A methylation of the long non-coding RNA (lncRNA) X-inactive-specific transcript (XIST) (31). ZC3H13 and CBLL1, in concert with additional cofactors, help to regulate nuclear m^6^A methylation (32). Furthermore, methyltransferase-like 16 (METTL16) was proposed to act as an independent mRNA methyltransferase in 2017 (33). It induces m^6^A modification in the 3’-UTR of mRNAs and at A43 in the U6 small nuclear RNA (snRNA), regulating tumorigenesis by targeting pre-mRNAs and non-coding RNAs (ncRNAs) (33, 34). Recently, it has been reported that methyltransferase-like 5 (METTL5) and zinc finger CCCH domain- containing protein 4 (ZCCHC4) are responsible for modifying 18S rRNA (35)and 28S rRNA (36), respectively. METTL5 is stabilized by transfer RNA (tRNA) methyltransferase 112 (TRMT112) (35). In addition, phosphorylated CTD- interacting factor 1 (PCIF1) mediates the N6, 2’-O-dimethyladenosine (m^6^Am) modification, an evolutionarily conserved mRNA modification (37). This involves catalyzing m^6^A methylation of 2-O-methylated adenines at the 5’ ends of mRNA (38). Furthermore, methyltransferase-like 4 (METTL4) has been identified as a novel internal m^6^Am methyltransferase that targets U2 snRNA, which regulates pre-mRNA splicing (39).

### Erasers

It was not until 2011 that scientists discovered that m^6^A modification could be reversed by RNA demethylases (also known as m^6^A erasers), which drew the attention of the broader academic community (40). These RNA demethylases include fat mass and obesity-associated protein (FTO) and AlkB homolog 5 (ALKBH5). ALKBH5 catalyzes the direct removal of m^6^A modifications, while FTO can sequentially oxidize m^6^A to N6-hydroxymethyladenosine (hm^6^A) and N6-formyladenosine (f^6^A), which are moderately stable and can later be hydrolyzed to adenine (40, 41). In addition, recent studies have shown that AlkB homolog 3 (ALKBH3) may serve as a novel demethylase that reverses m^6^A modifications (42). m^6^A-modified mammalian tRNA has been identified as a novel ALKBH3 substrate, highlighting a novel role for ALKBH3 in tumor progression *via* RNA demethylation and subsequent promotion of protein synthesis (43).

### Readers

M^6^A readers recognize and bind to m^6^A sites, altering the destinies of their target RNAs. They can also influence mRNA by destabilizing its structure, which can affect the binding of diverse RNA-binding proteins. Pull-down assays using a methylated probe and quantitative protein mass spectrometry assays have identified multiple m^6^A-binding proteins. The functions of m^6^A modification, which include regulating RNA splicing, export, degradation, stability and translation, are mainly exerted *via* the recruitment of these m^6^A readers. There are three classes of these proteins.

The class I m^6^A readers are YTH domain-containing proteins (YTH domain- containing family protein 1/2/3 [YTHDF1/2/3] and YTH domain-containing1/2 [YTHDC1/2]). YTHDF2 was the first identified and is the most studied m^6^A reader. Nuclear YTHDF2 binds to m^6^A sites in the 5’-UTR, prevents demethylation by FTO and thereby promotes cap-independent translation during the heat shock response (45). Cytoplasmic YTHDF2 promotes degradation of its target mRNAs partly by recruiting the CCR4-NOT deadenylase complex (46). Independently, YTHDF1 binding promotes the translation of m^6^A-modified mRNAs by recruiting the eukaryotic initiation factor 3 (eIF3) translation initiation complex (44). YTHDF3, in synergy with YTHDF1, facilitates translation and it also affects YTHDF2-mediated mRNA decay (47). All three YTHDF proteins function cooperatively in fundamental biological pathways (72). Moreover, nuclear YTHDC1 has been reported to regulate mRNA splicing, expedite mRNA export (48) and accelerate the decay of certain mRNAs (49). Additionally, this protein preferentially recognizes m^6^A residues in the lncRNA XIST and is required for XIST-mediated transcriptional silencing (31). Furthermore, YTHDC2 can be both nuclear and cytosolic (50–52). It was found to interact with RNA helicase to positively regulate translation elongation in an m^6^A-dependent manner (50). Additionally, YTHDC2 can mediate mRNA degradation by recruiting the 5′–3′ exoribonuclease XRN1 (51).

The class II m^6^A readers are three heterogeneous nuclear ribonucleoproteins (hnRNPs): hnRNPC, hnRNPG and hnRNPA2B1. These proteins can remodel the local RNA structure in an m^6^A-dependent manner and, consequently, modulate nearby RNA–protein interactions, in a mechanism known as an “m^6^A-switch”. hnRNPC regulates pre-mRNA processing and alternative splicing (53), modulating the alternative splicing of nearby exons (54). In contrast, hnRNPA2B1 is a nuclear reader that recognizes primary (pri)-miRNA m^6^A modifications and subsequently stimulates miRNA processing and alternative splicing (55, 56).

The class III m^6^A readers are insulin-like growth factor 2 mRNA-binding proteins (IGF2BPs), including IGF2BP1-3. These proteins are a distinct family of cytoplasmic m^6^A readers. They recognize the GG(m^6^A)C consensus sequence *via* their K homology (KH) domains and enhance the stability and translation of their target mRNAs under normal and stress conditions (57). These proteins function by recruiting RNA stabilizers, such as Hu antigen R (HuR), which is an indirect m^6^A effector with a preference for less m^6^A-modified transcripts, in order to maintain mRNA stability (58–60). Specifically, by promoting the expression of serum response factor (SRF) in an m^6^A-dependent manner *via* impairing the miRNA-dependent decay of the SRF mRNA, IGF2BP1 promotes SRF-dependent transcription in cancer, thereby enhancing tumor cell growth and invasion. At the post-transcriptional level, IGF2BP1 maintains the expression of multiple SRF target genes, including PDZ and LIM domain 7 (PDLIM7) and forkhead box K1 (FOXK1), which further enhances tumor cell growth and invasion (61). IGF2BP2 regulates the m^6^A-modified lncRNA DANCR to promote cancer stemness-like properties and cancer pathogenesis (62). Lastly, IGF2BP3 regulates RNA stability, RNA degradation, RNA localization and miRNA biogenesis, but the exact molecular processes underlying these functions have only begun to be elucidated (60, 63). Furthermore, several novel m^6^A readers have been identified, including fragile X mental retardation protein (FMRP), leucine-rich pentatricopeptide-repeat containing (LRPPRC), the eIF3 complex, staphylococcal nuclease domain-containing protein 1 (SND1), proline rich coiled-coil 2A (PRRC2A), and ribosomes. FMRP contains three KH domains and one arginine–glycine–glycine (RGG) domain and has been shown to prefer m^6^A-containing RNA, repressing target mRNA translation and maintaining target mRNA stability, likely by interacting with YTHDF1 and YTHDF2 (64, 65). LRPPRC is indispensable for maintaining the pool of non-translated transcripts and for regulation of mitochondrial translation (66, 67). The eIF3 complex is sufficient to recruit the 43S complex to initiate translation in the absence of the cap-binding factor eIF4E (68) and eIF3h also interacts with cytoplasmic METTL3 to bring about enhanced cap-dependent translation, the formation of densely packed polyribosomes and oncogenic transformation (28). SND1 is reported to be an m^6^A reader that stabilizes open reading frame 50 (ORF50) RNA and is essential for Kaposi’s sarcoma associated herpesvirus (KSHV) replication (69). PRRC2A plays an important role in oligodendrocyte specification by stabilizing m^6^A-modified oligodendrocyte lineage transcription factor 2 (Olig2) mRNA (70). Lastly, ribosomes may also act as m^6^A readers. Single-molecule ribosome translocation experiments have shown that an mRNA molecule containing a single-base m^6^A modification can change the translation dynamics by ribosomes in *Escherichia coli* (e.g., acting as a barrier to translation elongation) (71). However, the effects of this mechanism on the stability and/or translation of m^6^A-modified mRNA need to be further investigated.

In summary, the intricate interactions between m^6^A modifications and RNA-binding proteins can regulate mRNA expression at multiple levels. Further investigation of these proteins and their dynamic roles in cancer biology is necessary to deepen our understanding of RNA methylation. The findings may provide novel insights into the mechanisms of cancer pathogenesis and potential therapeutic strategies.

## Implications of m^6^A Modification in HCC

**Figure 2:**
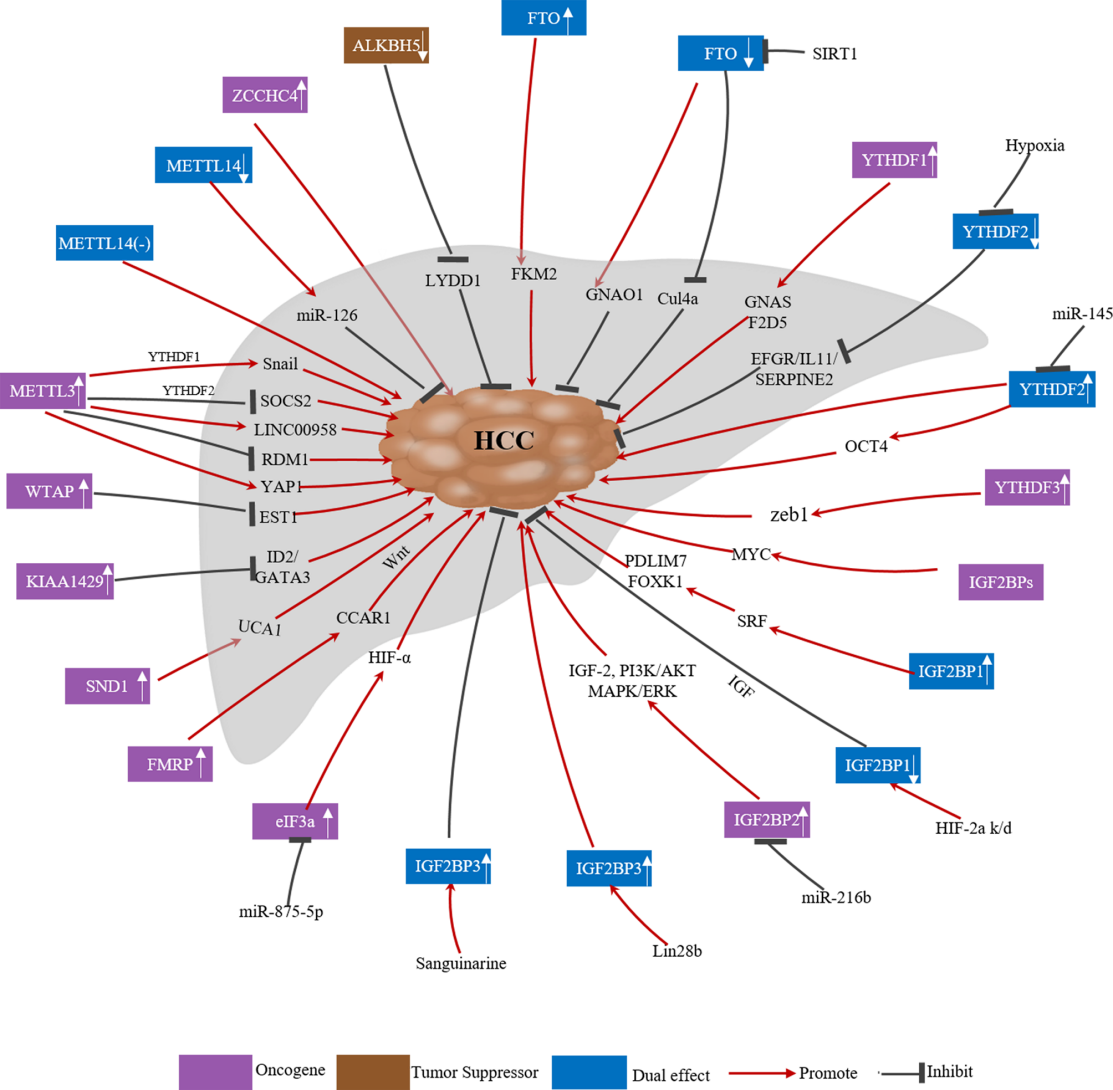
Deregulation of m6A related regulators in human HCC.

Owing to advances in RNA sequencing, a growing number of studies have shown that m^6^A modification plays an important role in human cancer progression. Genes encoding m^6^A-related regulators can act as oncogenes or tumor suppressor genes. The expression of these regulators can be affected by various factors, such as tumor heterogeneity, the presence of hypoxic microenvironments and post-translational modifications, which affect their function in cancer. The specific roles of m^6^A-related regulators in HCC are summarized in **Figure 2** and **Table 2**.

**Table 2:** Roles of m^6^A-related regulators in HCC.

Regulator	Role	Trend	Target	Mechanisms	Functions	Refs
METTL3	Oncogene	Up	SOCS2	Promotes SOCS2 degradation in an m6A-YTHDF2-dependent manner	Increases tumorigenicity, growth and lung metastasis	(19)
METTL3	Oncogene	Up	Snail	Regulates key EMT translator Snail through m6A-YTHDF1 pathway	Increases cancer cell migration, invasion and EMT	(73)
METTL3	Oncogene	Up	YAP1	Facilitates the translation of YAP1 mRNA	Increases the formation of vasculogenic mimicry in HCC	(74)
METTL3	Oncogene	Up	LINC00958	Increases LINC00958 expression and stability, which sponges miR-3619-5p to upregulate HDGF	Increases HCC lipogenesis and progression	(75)
METTL3	Oncogene	Up	RDM1	Reduces RAD52 motif 1 (RDM1) mRNA expression	Increases proliferation, colony formation and cell population at G2/M phase	(76)
METTL3	Oncogene	Up	Snail	SUMOylated by the small ubiquitin-like modifier SUMO	Increases HCC progression	(77)
METTL14	Suppressor	Down	miR-126	Interacts with DGCR8 and modulates the primary miR-126 process	Inhibits tumor invasion and metastasis	(17)
METTL14	Oncogene	Unchanged	–	–	Promotes HCC cell proliferation and migration	(19)
WTAP	Oncogene	Up	ETS1	Suppresses ETS proto-oncogene 1 (ETS1) in an m6A-HuR-dependent manner	Increases proliferative capacity	(78)
KIAA1429	Oncogene	Up	GATA3	Inhibits HuR binding to GATA3 pre-mRNA and promotes degradation of the pre-mRNA	Increases HCC growth and metastasis	(79)
KIAA1429	Oncogene	Up	ID2	Inhibits ID2 mRNA	Increases HCC growth and metastasis	(80)
ZCCHC4	Oncogene	Up	–	–	Increases cell proliferation and tumor size in a xenograft mouse model	(81)
FTO	Oncogene	Up	PKM2	Increases PKM2 expression	Increases proliferation and tumor growth	(82)
FTO	Suppressor	Down	GNAO1	Increases tumor suppressor GNAO1 mRNA expression	Inhibits HCC tumorigenesis	(83)
FTO	Suppressor	Down	Cul4a	Inhibits Cul4a translation	Inhibits HCC cell cycle progression and proliferation	(84)
ALKBH5	Suppressor	Down	LYPD1	Reduces LYPD1 stability mediated by m6A reader IGF2BP1	Inhibits HCC cell proliferation and invasion *in vitro and in vivo*	(85)
ALKBH3	Oncogene	Up	–	Affects cell cycle through p21/p27 pathway	Increases HCC cell proliferation *in vitro* and *in vivo*	(86)
YTHDF1	Oncogene	Up	GNAS	Increases GNAS translation	Increases LPS-induced HCC cell growth and invasion	(87)
YTHDF1	Oncogene	Up	FZD5	Accelerates the translational output of FZD5	Increases HCC cell proliferation and metastasis *in vitro and in vivo*	(88)
YTHDF1	Oncogene	Up	AKT/GSK3β/β-catenin	Facilitates EMT and AKT/GSK3β/β-catenin signaling	Increases HCC progression	(21)
YTHDF2	Oncogene	Up	–	Negatively regulated by miR-145	Increases HCC cell proliferation	(89)
YTHDF2	Oncogene	Up	OCT4	Increases OCT4 expression	Increases liver cancer stem cell phenotype and cancer metastasis	(90)
YTHDF2	Suppressor	Down	EGFR	Increases EGFR degradation	Inhibits HCC cell proliferation and tumor growth	(91)
YTHDF2	Suppressor	Down	IL11 and SERPINE2	Increases decay of IL11 and SERPINE2	Inhibits inflammation, vascular reconstruction and metastatic progression	(92)
YTHDF3	Oncogene	Up	Zeb1	Increases Zeb1 mRNA stability	Increases HCC cell migration, invasion and EMT	(93)
IGF2BPs	Oncogene	Up	MYC	Increases MYC, FSCN1 and TK1 stability	Increases HepG2 cell proliferation, migration/invasion and colony formation	(59)
IGF2BP1	Oncogene	Up	SRF	Impairs SRF decay and subsequently promotes PDLIM7 and FOXK1 translation	Increases tumor cell growth and invasion	(61)
IGF2BP1	Suppressor	–	–	Decreased by HIF-2α k/d and interfered with IGF signaling	Silencing IGFBP1 increases the potential of HepG2 cells to induce lymphangiogenesis	(94)
IGF2BP2	Oncogene	Up	–	Promotes the integrin β1/FAK/Erk/Elk1/EGR1 pathway	Increases HCC cell proliferation	(95)
IGF2BP2	Oncogene	Up	–	Negatively regulated by miR-216b and activates the downstream IGF2/PI3K-AKT/MAPK-ERK signaling pathways	Increases cell proliferation *in vitro and in vivo*	(96)
IGF2BP3	Oncogene	Up	–	Acts as an oncogene downstream of Lin28b	Increases growth in murine models	(97)
IGF2BP3	Suppressor	Up	–	Upregulated by sanguinarine	Increases Hep3B cell apoptosis and inhibits proliferation	(98)
IGF2BP3	Suppressor	–	–	Suppresses transcription of EGR1 and its target genes bFGF and PDGF	Inhibits HCC cell proliferation	(99)
eIF3a	Oncogene	Up	HIF1α	Regulates glycolysis through HIF1α IRES-dependent translation	Increases HIF1-α protein level and cellular glycolysis ability	(100)
eIF3a	Oncogene	Up	–	Negatively correlated with miR-875-5p	Increases HCC cell proliferation, motility and EMT	(101)
FMRP	Oncogene	Up	CCAR1	Binds to CCAR1 and assists in the activation of the Wnt/β-catenin pathway	Increases cellular stemness	(102)
SND1	Oncogene	Up	UCA1	Increases lncRNA UCA1 expression *via* a transcriptional activator MYB	Inhibits apoptosis in HCC cells	(103)

### Writers and HCC

As the most important component of the m^6^A MTC, the role of METTL3 in HCC has been widely reported. METTL3 is upregulated in HCC and high METTL3 expression is associated with poor HCC prognosis. It can promote the growth, migration and invasion of cancer cells both *in vitro* and *in vivo*, serving as an oncogene through various mechanisms (19, 20, 73, 74). First, Chen et al. reported that METTL3 facilitates HCC tumorigenicity, growth and lung metastasis *in vivo* in an m^6^A-YTHDF2-dependent manner by promoting the degradation of suppressor of cytokine signaling 2 (SOCS2) mRNA (19). Second, Lin et al. found that METTL3 regulates cancer cell epithelial–mesenchymal transition (EMT) by upregulating Snail (a key transcription factor in EMT) through the m^6^A-YTHDF1 pathway (73). Third, Zuo et al. showed that METTL3 enhances the expression and stability of LINC00958, which targets miR-3619-5p in order to upregulate hepatoma-derived growth factor (HDGF), thereby facilitating HCC lipogenesis and progression (75). Fourth, Chen et al. demonstrated that METTL3 overexpression markedly reduces RAD52 motif 1 (RDM1) mRNA expression *via* m^6^A modification; RDM1 knockdown increases HCC cell proliferation, colony formation and the cell population at the G2/M phase, as RDM1 serves as a tumor suppressor in HCC (76). Fifth, Lin et al. reported that METTL3 is involved in the regulation of glycolysis in HCC *via* regulating mTORC1 activity (104). Sixth, Qiao et al. found that METTL3 promotes vasculogenic mimicry in HCC *via* the Hippo pathway by facilitating the translation of Yes-associated protein 1 (YAP1) mRNA (74). Seventh, Xu et al. demonstrated that METTL3 can be SUMOylated by the small ubiquitin-like modifier SUMO and subsequently controls Snail mRNA homeostasis in an m^6^A methyltransferase activity-dependent manner, thus promoting HCC progression (77). Moreover, METTL3 is significantly downregulated in human sorafenib-resistant HCC. Depletion of METTL3 in mouse xenograft models increases sorafenib resistance in HCC by decreasing the stability of FOXO3 mRNA (without METTL3 depletion, METTL3 causes m^6^A modification of FOXO3 mRNA, increasing its stability *via* a YTHDF1-dependent mechanism) (105).

Similarly, WTAP is highly expressed in HCC tissue and is an independent predictor of survival in HCC patients (78, 106). WTAP suppresses ETS proto-oncogene 1 (ETS1) in an m^6^A-HuR-dependent manner, which upregulates the cell cycle regulators p21 and p27 to promote the G2/M phase in HCC cells, thus increasing proliferation (78). KIAA1429 is elevated in HCC and high KIAA1429 expression is associated with poor HCC prognosis (79, 80). It promotes HCC cell migration and invasion by altering the m^6^A modification of inhibitor of DNA binding 2 (ID2) and GATA binding protein 3 (GATA3) mRNA (79, 80). Relatedly, circ_KIAA1429 (hsa_ circ_0084922) was upregulated in HCC cells and tissues. Circ_KIAA1429, which maintains zinc finger E-box binding homeobox 1 (ZEB1) expression *via* an m^6^A-YTHDF3-ZEB1 mechanism in HCC, can facilitate HCC cell migration, invasion and EMT (93). Furthermore, RBM15B was also reported to be dramatically upregulated in human HCC (19).

Unlike other m^6^A methyltransferases, METTL14 was downregulated in HCC, and low METTL14 protein expression is associated with shorter overall survival (OS) and recurrence-free survival (17, 92, 107). Ma et al. (17) reported that METTL14 suppressed HCC invasion and metastasis by interacting with the micro-processor protein DiGeorge syndrome key region gene 8 (DGCR8) and positively promoting primary miR-126 maturation in an m^6^A-dependent manner. Likewise, Liu et al. (108) and Li et al. (109) analyzed data from The Cancer Genome Atlas (TCGA) and Gene Expression Omnibus (GEO) databases and found that METTL14 was significantly downregulated in HCC. METTL14 may inhibit HCC progression by altering the m^6^A modification of cysteine sulfonic acid deacidiase (CSAD), glutamic-oxaloacetic transaminase 2 (GOT2) and SOCS2 (109). In contrast, Chen et al. (19) reported that METTL14 was unchanged in HCC in their study, but METTL14 knockdown significantly suppressed Huh-7 cell proliferation, migration and colony formation (19).

Given the paradoxical expression patterns and roles of METTL14 in prior studies of HCC, Zhang et al. (110) analyzed paired HCC and normal samples in multiple microarray datasets. Unexpectedly, their results indicated that METTL14 may have a variety of roles as opposed to only being a tumor suppressor or promoter in hepatocarcinogenesis. They ascribed the contradictory findings regarding the METTL14 expression pattern and role in HCC samples compared to normal samples to the heterogeneity of HCC cell lines and clinical samples, the versatility of the METTL3-METTL14 heterocomplex, and m6A-independent effects of METTL14. Additionally, METTL16 is a newly identified RNA methyltransferase that operates independently of the MTC. Wang et al. (111) carried out a bioinformatics analysis using TCGA data and reported that METTL16 was downregulated in HCC and that low METTL16 expression was associated with poor OS and disease-free survival. Another new m^6^A methyltransferase, ZCCHC4, was overexpressed in HCC, and ZCCHC4 knockout eliminated m^6^A modification of 28S rRNA, inhibited HepG2 cell proliferation and reduced the tumor size in a xenograft mouse model (81).

In summary, these results reveal that m^6^A writers, including those in the MTC, regulate HCC progression. Except for METTL16 and METTL14, the m^6^A writers are upregulated in liver cancer tissues and act as oncogenes. Further investigations are required to resolve the contradictions.

### Erasers and HCC

The expression and role of FTO in liver cancer is controversial. Li et al. (82) reported that upregulated FTO was associated with poor HCC prognosis and demonstrated that FTO can induce HCC tumorigenesis by regulating pyruvate kinase M2 (PKM2) in an m^6^A modification-dependent manner. However, in addition to its oncogenic function, FTO also suppresses HCC progression. Ma et al. (17) and Hou et al. (92) reported that FTO was downregulated in liver cancer tissues. More specifically, Ma et al. (17) observed that METTL14 and FTO were downregulated in cancer tissues compared to adjacent tissues and there was a correlation between them. Although both proteins are expressed in the nucleus, there is little interaction; METTL14 silencing or overexpression had no significant effect on FTO expression. Despite this, Ma et al. suggested that the eventual FTO downregulation in liver cancer with reduced METTL14 expression may involve a compensatory feedback mechanism. Later, Liu et al. (83) reported that FTO is negatively regulated by the deacetylase SIRT1 *via* RANBP2-mediated SUMOylation; its downregulation reduces the expression of its target gene guanine nucleotide-binding protein G(O) subunit alpha (GNAO1), thereby promoting the progression of liver cancer. Moreover, a subsequent study revealed that FTO might target *Cul4a* mRNA to downregulate CUL4A protein, thereby presumably blocking HCC cell cycle progression and proliferation (84).

ALKBH5, another m^6^A demethylase, was downregulated in HCC, and decreased ALKBH5 expression was an independent prognostic factor associated with poor survival in HCC patients (85). ALKBH5-mediated m^6^A demethylation suppresses proliferation and invasion by suppressing IGF2BP1-mediated LY6/PLAUR domain-containing 1 (LYPD1) RNA stability (85). Additionally, Liu et al. (112) reported that ALKBH5 inhibits autophagy in sorafenib-treated HCC cells and can be inhibited by an important circRNA known as cIARS (hsa_circ_0008367). Moreover, recent research has identified another m^6^A demethylase, ALKBH3 (43). Wang et al. (86) reported that ALKBH3 was overexpressed in HCC and high ALKBH3 expression in HCC tissues decreased OS and disease-free survival in HCC patients. ALKBH3 knockdown inhibited human HCC cell proliferation *in vitro* and xenograft tumor formation *in vivo*, presumably through p21/p27-mediated cell cycle arrest at the G1 phase (86).

In summary, these results suggest that m^6^A erasers are involved in regulating HCC progression, but some of the conclusions are contradictory, suggesting a complex role of m^6^A modification in HCC.

### Readers and HCC

Like writers and erasers, multiple m^6^A readers have also been implicated in liver cancer. Many studies have reported upregulation of YTHDF1 in liver cancer tissues and its overexpression is associated with poor HCC prognosis. It promotes HCC cell proliferation and metastasis both *in vitro* and *in vivo* (21, 88, 113–115). Regarding the underlying mechanism, Ding et al. (87) found that high expression of G-protein alpha-subunit (GNAS) promotes lipopolysaccharide-induced HCC cell growth and invasion by interacting with signal transducer and activator of transcription 3 (STAT3) in an m^6^A-YTHDF1-dependent manner. Additionally, Lin et al. (73) reported that YTHDF1 mediates m^6^A-induced translation of Snail mRNA, which triggers polysome-mediated translation and regulates EMT in cancer cells. Bian et al. (21) identified YTHDF1 as an HCC oncogene that facilitates EMT and AKT/GSK3β/β-catenin signaling. Liu et al. (88) revealed that YTHDF1 can act as an oncogene by mediating the m^6^A-dependent acceleration of the translation of FZD5 mRNA, which is involved in the WNT/β-catenin pathway. Nevertheless, there are conflicting opinions on YTHDF1 expression in liver cancer tissues, as Hou et al. (92) assessed YTHDF1 mRNA levels in 51 paired HCC and paracancerous tissues by qRT-PCR and found no significant difference between the two tissue types. Furthermore, the role of YTHDF2 in liver cancer is controversial. Yang et al. (89) found that miR-145 modulates m^6^A levels by targeting the 3’-UTR of YTHDF2 mRNA in HCC cells. As miR-145 is frequently downregulated in HCC and targets YTHDF2, upregulated YTHDF2 in HCC appears to be closely related to the malignancy of HCC. Additionally, Zhang et al. (90) reported that YTHDF2 was a predictor of poor HCC prognosis and it promotes the liver cancer stem cell phenotype and metastasis by upregulating octamer-binding transcription factor-4 (OCT4) in an m^6^A-dependent manner. Nevertheless, YTHDF2 may also act as a tumor suppressor, as Zhong et al. (91) and Hou et al. (92) reported that it was downregulated by hypoxia in HCC. The former study found that YTHDF2 directly binds to the m^6^A modification site of EGFR mRNA 3’-UTR to promote mRNA degradation, thereby suppressing HCC cell proliferation and tumor growth (91). The latter study found that YTHDF2 was downregulated in HCC by hypoxia-inducible factor (HIF)-2α, which reduced the degradation of m^6^A-containing interleukin 11 (IL11) and serpin family E member 2 (SERPINE2) mRNAs, leading to inflammation-mediated malignancy and disruption of vascular normalization (92). Moreover, another YTH domain protein, YTHDF3, is upregulated in HCC and increases ZEB1 mRNA stability in an m^6^A-dependent manner (93). ZEB1 is the downstream target of circ_KIAA1429 and its upregulation leads to HCC metastasis. In summary, these contradictory functions may be related to the tumor heterogeneity and/or the small sample sizes used.

The roles of the three hnRNPs, which are class II m^6^A readers, in liver cancer have rarely been studied. However, trichostatin A-induced lncRNA-uc002mbe.2 directly bound to hnRNPA2B1 and promoted its degradation. This hnRNPA2B1 downregulation contributed to AKT deactivation and p21 upregulation, resulting in liver cancer cell apoptosis and the inhibition of proliferation *in vitro* and *in vivo* (116).

Regarding class III m^6^A readers, IGF2BPs are highly expressed in HCC, playing oncogenic roles in HepG2 cells by enhancing MYC mRNA stability and post-transcriptionally upregulating target gene expression (59). Consistently, IGF2BP1 upregulates SRF in an m^6^A-dependent manner by decreasing the miRNA-mediated decay of SRF mRNA, which subsequently leads to increased PDLIM7 and FOXK1 translation. This results in tumor cell growth and invasion, leading to a poor OS in liver cancer patients (61). Gong et al. (117) reported that inhibition of fatty acid synthase (FASN) decreases IGF2BP1 expression, along with HIF-1α activity, thereby suppressing HCC cell migration and invasion. Moreover, He et al. (118) demonstrated that IGF2BP1 interacts with glioma-associated oncogene homologue 1 (GLI1) mRNA, which is involved in HCC progression; the liver-specific lncRNA LINC01093 disrupts this interaction. The above studies suggest that IGF2BP1 upregulation plays an oncogenic role. However, it has also been reported that IGF2BP1 downregulation can promote tumor progression (94, 119). Geis et al. (94) identified and verified IGFBP1 as a target gene of the transcription factor HIF-2α, and silencing of IGF2BP1 significantly enhanced the potential of HepG2 cells to induce lymphangiogenesis. In addition, Nielson et al. (119) reported that hepatitis B virus (HBV) suppresses IGF2BP1 secretion to facilitate pro-survival and anti-apoptotic insulin-like growth factor (IGF)-1 activity. Adding recombinant IGF2BP1 reversed the anti-apoptotic effect in HepG2 cells.

Regarding IGF2BP2, it was upregulated in HCC patients compared to healthy controls, and IGF2BP2 expression was positively associated with decreased tumor differentiation and increased size, metastasis and portal vein infiltration in HCC (95). Moreover, exogenous IGF2BP2 promoted the integrin β1/FAK/Erk/Elk1/EGR1 pathway, which stimulated HCC cell proliferation (95). Furthermore, Liu et al. (96) showed that HBV X protein (HBx) in HepG2 cells downregulated miR-216b and thereby upregulated IGF2BP2, which activated the downstream insulin-like growth factor 2 (IGF-2), PI3K/AKT and MAPK/ERK signaling pathways, thus promoting cell proliferation and invasion. Regarding IGF2BP3, it has been reported to be a downstream oncogenic effector of LIN28B, whose overexpression drives liver tumorigenesis in murine models (97). In contrast, Wang et al. (98) reported that sanguinarine can upregulate IGF2BP3 in Hep3B cells and thereby promote apoptosis. Similarly, it was shown that IGFBP3 can inhibit HCC cell proliferation by suppressing transcription of early growth response protein 1 (EGR1) and its target genes basic fibroblast growth factor (bFGF) and platelet-derived growth factor (PDGF) (99).

Increasing numbers of new m^6^A readers are being identified. In eukaryotes, the eIF3 complex, composed of 13 subunits from eIF3a to eIF3m, is the largest and most complex translation initiation factor. eIFs play major roles in the initiation step of protein translation (100). M^6^A modifications in the 5′-UTR of transcripts can directly recruit eIF3, contributing to the assembly of translation initiation complexes on eIF3-specialized mRNAs (68). Golob-Schwarzl et al. (120) reported that various eIF3 subunits were significantly increased in chronic HBV-associated HCC. Recently, eIF3a has been recognized as a proto-oncogene, which is overexpressed in HCC and linked to HCC tumorigenesis (100, 101, 121). Heo et al. (121) reported that eIF3a is significantly upregulated in HCC tissue compared to normal tissue in mice and patients and combined detection of anti-eIF3a autoantibody and alpha-fetoprotein (AFP) in patient sera improved the accuracy of HCC diagnosis. Additionally, Miao et al. (100) found that eIF3a regulates cellular glycolysis by increasing HIF-1α protein expression *via* internal ribosomal entry site (IRES)-dependent translation and eIF3a predicts poor HCC prognosis. Moreover, Chen et al. (101) demonstrated that eIF3a had an oncogenic role and was upregulated and negatively correlated with miR-875-5p expression in HCC tissues; eIF3a knockdown inhibited HCC cell proliferation, motility and EMT. Similarly, Yue et al. (122) revealed that eIF3b was upregulated in liver cancer tissues and it had promising prognostic value, as high eIF3b expression was generally associated with shorter OS and relapse-free survival. Lastly, eIF3c has also been reported to be an oncogene in liver cancer. Li et al. (123) found that eIF3c was upregulated during HCC progression and associated with poor survival in TCGA datasets. Lee et al. (124) also reported that the expression of eIF3c in HCC cells significantly increased extracellular exosome secretion and these eIF3c-enhanced exosomes were oncogenic and potentiated tumor angiogenesis.

FMRP also plays an oncogenic role in HCC. Recently, Zhu et al. (102) reported that FMRP increases cellular stemness in HCC *via* its target gene Cell division cycle and apoptosis regulator 1 (CCAR1), which assists in Wnt/β-catenin pathway activation. Additionally, SND1, a subunit of the RNA-induced silencing complex (RISC), has been implicated as an oncogene in HCC (103, 125, 126). First, SND1 can regulate cellular cholesterol distribution and homeostasis in HCC cells (126) and promote tumor-initiating cell (TIC) formation *via* the Akt and NF-κB signaling pathways in Alb/SND1 mice (125). Second, Cui et al. (103) analyzed Human Protein Atlas (HPA) and TCGA data and found that SND1 was significantly upregulated in liver cancer patients. SND1 is an anti-apoptotic factor in HCC cells and positively regulates lncRNA UCA1 expression *via* the transcriptional activator MYB (103). LRPPRC, a novel regulator of m6A modification, was revealed has prognostic value in HCC patients. High levels of LRPPRC are beneficial to the OS, but the precise molecular mechanisms remain elusive (127).

To sum up, m^6^A modification can affect the fate of mRNAs by recruiting m^6^A readers. The various m^6^A readers have different effects on HCC. However, the roles of YTHDF2, IGF2BP1 and IGF2BP3 in HCC are controversial and more studies are needed to further explore these factors.

## Clinical Applications of m^6^A Modification in HCC

As RNA methylation plays an extensive regulatory role in HCC, RNA methylation profiling has the potential to be used as a clinical tool. The diagnosis and prognostic value of m^6^A-related regulators has been reported in several studies. METTL3 and YTHDF1 are both overexpressed in HCC in several studies. As METTL3/YTHDF1 overexpression is associated with poor HCC prognosis, they may be prognostic markers and therapeutic targets (19–21). Additionally, KIAA1429 was considerably upregulated in HCC tissues, and high KIAA1429 expression was associated with poor HCC prognosis (79). Another study found that eIF3b was highly expressed in liver cancer tissues and had promising diagnostic and prognostic value (122).

In addition to having diagnostic and prognostic value, m^6^A modification and m^6^A-related regulators may also be useful for developing treatments. Recently, progress has been made regarding experimental therapies targeting m^6^A-related mechanisms. The natural product rhein was the first identified FTO inhibitor; it exerts good inhibitory activity against FTO and increases m^6^A modification levels (128). However, it is not a selective FTO inhibitor, as it can also bind to a different part of the active site in AlkB than it binds to in FTO (129). Later, R-2-hydroxyglutarate (R-2HG) (130) and meclofenamic acid (MA) (131) were identified as FTO inhibitors and confirmed to inhibit tumor cell growth and induce apoptosis. Lu et al. (132) reported that curcumin can affect METTL3, METTL14, ALKBH5, FTO and YTHDF2 expression and subsequently increase m^6^A modification, thereby inhibiting lipopolysaccharide-induced liver injury and lipid metabolism disorders in piglets. Hou et al. (92) demonstrated that YTHDF2 downregulation in HCC is regulated by HIF-2α, and an HIF-2α antagonist (PT2385) can upregulate YTHDF2 *in vitro* (without changing its cytosolic distribution) and repress liver cancer. Furthermore, to treat HCC, Zuo et al. (75) developed novel PEGylated poly (lactic-co-glycolic acid) nanoparticles (PLGA-PEG NPs) loaded with si-LINC00958 targeting LINC00958, which is otherwise upregulated and stabilized by METTL3-mediated m^6^A modification, which promotes HCC progression. The PLGA-PEG NPs exhibited controllable drug release, excellent cellular uptake and precise tumor-targeting capacity. Moreover, tumor growth was significantly inhibited and the OS was remarkably prolonged in HCC-bearing mice injected with the PLGA-PEG NPs. Additionally, the pathological and blood test results indicated that there were no significant adverse effects in the mice. Lastly, Wang et al. (98) reported that sanguinarine can upregulate IGF2BP3 and thereby promote Hep3B cell apoptosis and inhibit proliferation, invasion and migration.

These studies suggest that HCC-related changes in the expression of m^6^A-related regulators may allow them to be used as HCC biomarkers and therapeutic targets. However, more studies are needed to determine the value of m^6^A-related regulators in early HCC diagnosis and the prediction of HCC prognosis and to assess their potential as therapeutic targets.

## Conclusions and Perspectives

In summary, increasing attention has been paid to the roles of m^6^A modification and dysregulated m^6^A-related regulators in HCC. In this review, we focused on the function of m^6^A RNA modification in HCC. Some of the studies discussed have reported contrasting results on the expression patterns or functions of the various m^6^A-related regulators. For example, most of the proteins that comprise the MTC are upregulated and promote HCC progression; however, METTL14 is decreased and can suppress liver cancer cell metastasis. Interestingly, an m^6^A-associated regulator can perform multiple biological functions *via* various target genes in HCC. The roles of FTO, YTHDF2, IGF2BP1 and IGF2BP3 in liver cancer are controversial. Some studies have reported that they are highly expressed in tumor tissues and are oncogenes (61, 82, 89, 90, 97). However, other studies have reported that they are downregulated in tumor tissues and serve as tumor suppressor genes (83, 84, 91, 92, 94, 98). The contradictory results may be related to cancer heterogeneity, cell background, the targeting specificity of the m^6^A-related regulators and small sample sizes. In addition, some researchers have explored targeted HCC therapies related to m^6^A modification. For example, the HIF-2α antagonist PT2385 can upregulate YTHDF2, thus inhibiting liver cancer, while si-LINC00958-loaded PLGA-PEG NPs exhibited controllable release, excellent cellular drug uptake and precise tumor-targeting capacity in HCC. However, few studies have focused on such targeted cancer therapies.

In the future, we intend to conduct the following research (1): screen for gene expression that can be used for early diagnosis and prognosis using a large sample of liver cancer cases (2), elucidate the mechanisms underlying the conflicting effects of different/the same m^6^A-related regulators in liver cancer and (3) develop specific inhibitors of m^6^A-related regulators for treating liver cancer. It is exciting that the recent development of m^6^A sequencing and editing tools will greatly facilitate m^6^A research at the single-nucleotide level, thus advancing the field.

## Author Contributions

NQ drafted the manuscript. XB constructed the figures. LM,QZ, and XX constructed the tables. FW, FH, and YS collected the references. BL revised the manuscript. XZ managed the article design, reviewed the manuscript, and provided funding support. All authors contributed to the article and approved the submitted version.

## Funding

This study was supported by grants from the National Natural Science Foundation of China (81802478) and Guangxi Natural Science Foundation (2018GXNSFBA050062).

## Conflict of Interest

The authors declare that the research was conducted in the absence of any commercial or financial relationships that could be construed as a potential conflict of interest.

## Publisher’s Note

All claims expressed in this article are solely those of the authors and do not necessarily represent those of their affiliated organizations, or those of the publisher, the editors and the reviewers. Any product that may be evaluated in this article, or claim that may be made by its manufacturer, is not guaranteed or endorsed by the publisher.

## Abbreviations

RBM15/15B, RNA-binding motif protein 15; CBLL1/HAKAI, Cbl proto-oncogene-like 1; METTL16, methyltransferase-like 16; METTL5, methyltransferase-like 5; ZCCHC4, zinc finger CCCH domain-containing protein 4; ALKBH3, AlkB homolog 3; hnRNP, heterogeneous nuclear ribonucleoprotein; IGF2BP, Insulin-like growth factor 2 mRNA-binding protein; HuR, Hu antigen R; FMRP, fragile X mental retardation protein; SND1, Staphylococcal nuclease domain-containing protein 1; PRRC2A, proline rich coiled-coil 2A; LRPPRC, leucine-rich pentatricopeptide-repeat containing.
